# Comparison of ultrasound-guided transversus abdominis plane block, quadratus lumborum block, and caudal epidural block for perioperative analgesia in pediatric lower abdominal surgery

**DOI:** 10.3906/sag-1812-59

**Published:** 2019-10-24

**Authors:** Celal Bulut İPEK, Deniz KARA, Sinan YILMAZ, Serdar YEŞİLTAŞ, Asım ESEN, Shainaaz Su Sandar Lwin DOOPLY, Kazım KARAASLAN, Ayda TÜRKÖZ

**Affiliations:** 1 Department of Anesthesiology and Reanimation, Faculty of Medicine, Bezmialem Vakıf University, İstanbul Turkey

**Keywords:** Pediatric surgery, transversus abdominis plane block, quadratus lumborum block, caudal epidural block, ultrasound-guided

## Abstract

**Background/aim:**

Despite different regional anesthesia techniques used to provide intraoperative and postoperative analgesia in pediatric patients, the analgesic effectiveness of peripheral nerve blockades with minimal side effect profiles have not yet been fully determined.We aimed to compare the efficacy of ultrasound-guided transversus abdominis plane (TAP) block, quadratus lumborum (QL) block, and caudal epidural block on perioperative analgesia in pediatric patients aged between 6 months and 14 years who underwent elective unilateral lower abdominal wall surgery.

**Materials and methods:**

Ninety-four patients classified under the American Society of Anesthesiologists physical status classification system as ASA I or ASA II were randomly divided into 3 equal groups to perform TAP, QL or Caudal epidural block using 0.25% of bupivacaine solution (0.5 ml kg−1).

**Results:**

Postoperative analgesic consumption was highest in the TAP block group (P < 0.05). In the QL block group, Pediatric Objective Pain Scale (POAS) scores were statistically significantly lower after 2 and 4 h (P < 0.05). The length of hospital stay was significantly longer in the caudal block group than the QL block group (P < 0.05).

**Conclusion:**

We suggest that analgesia with ultrasound-guided QL block should be considered as an option for perioperative analgesia in pediatric patients undergoing lower abdominal surgery if the expertise and equipment are available.

## 1. Introduction

Recently, regional anesthesia techniques have been replaced by peripheral nerve blocks in the management of perioperative pain. Because of the widespread use of ultrasonography, it has been reported that peripheral nerve blocks showed similar analgesic efficacy with favorable rates of side effects when compared to central blocks.

Central nerve blocks are often used in combination with general anesthesia for pediatric surgery in order to reduce general anesthetic requirements, opioid use, postoperative pain, nausea and vomiting, and risk of anesthetic neurotoxicity, particularly in young patients [1–4]. Caudal epidural block (CEB) is a well-established and commonly performed neuraxial technique for providing intraoperative and postoperative analgesia in pediatric patients scheduled for lower abdominal perineal surgical interventions [5–6]. Although the efficacy and safety of CEB are fairly high [7], the associated complications such as inadvertent dural puncture, unwarranted motor blockade of lower limbs, and disturbance of bladder function [8] might limit its use.

Undoubtedly, introduction of ultrasonography into anesthesia practice has led to an increase in practice of peripheral nerve blocks. Ultrasonography guidance has significantly facilitated the practice of regional nerve blockades [9]. There has been a growing interest in ultrasound-guided transversus abdominis plane (TAP) block as an alternative and valid postoperative analgesic method in pediatric patients undergoing lower abdominal surgery [10].

Quadratus lumborum block (QL block) is a new abdominal and truncal block used for providing somatic analgesia of both upper and lower abdominal pain which was described by Blanco as a posterior variant of the TAP block [11].

As the effectiveness and minimal adverse effect profiles of perineural blockade in pediatric surgery were not yet fully determined, we conducted this randomized controlled trial to investigate the efficacy and safety of ultrasound-guided TAP block, QL block, and CEB for perioperative analgesia on pediatric patients during the first 24 h following unilateral lower abdominal surgery.

## 2. Methods

After obtaining approval of the Ethical Board of the Bezmialem Vakıf University (Ethical Committee N: 71306642-050.01.04), we found 94 pediatric patients aged 6 months to 14 years, with American Society of Anesthesiologists (ASA) physical status of I or II, who were scheduled for elective unilateral lower abdominal surgery under general anesthesia to include in our study. Informed consent was obtained from the family of each patient. Parents and a second anesthetist were blinded to group assignment in the recovery room and the surgical ward.

Patients with known allergies to local anesthetics, infection of injection sites, coagulation disorders, liver–kidney diseases, or unwillingness to participate were excluded from the study.

Patients were premedicated with oral midazolam 0.5 mg kg−1 30 min before surgery. After standard anesthesia monitorization including electrocardiogram, heart rate, noninvasive blood pressure, peripheral oxygen saturation (SpO2), end-tidal carbon dioxide, temperature, and Bispectral index (BIS), anesthesia was induced with inhalation of 8% sevoflurane in 50% air in oxygen under spontaneous ventilation. Afterwards, peripheral venous access was established to administer propofol 2 mg kg−1 and fentanyl citrate 1 μg kg−1. Laryngeal mask airway was used to secure the upper airway. Anesthesia was maintained with sevoflurane in 50% air in oxygen by targeting BIS scores of 50–60 in all groups. During the operation, a fentanyl infusion of 0.5 μg kg−1 was administered if the blood pressure and heart rate increased to 20% higher than the baseline value. An isotonic balanced electrolyte solution of 10 ml kg−1 h−1 was administered intravenously throughout the surgery.

All the blocks were performed by the same anesthesiologist after the placement of laryngeal mask airway before onset of surgery under ultrasound guidance (Zonare Inc., Mountain View, CA, USA).

### 2.1.1. TAP block

**Figure 1:**
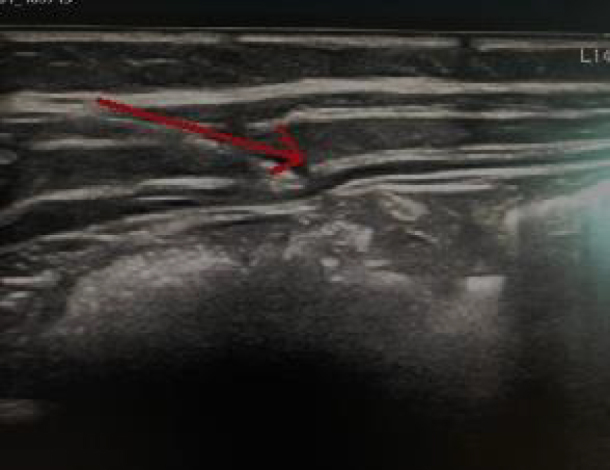
TAP block.

After induction of anesthesia at the supine position, a 14-5 MHz linear ultrasound probe was placed between the anterolateral abdominal wall and iliac crest. The external abdominal oblique, internal abdominal oblique, and transversus abdominis muscles were identified using the probe. A 22-gauge, 50-mm needle was inserted using the in-plane technique. The needle was advanced until it reached the neurofascial plane between the internal oblique and transversus abdominis muscles. After careful aspiration to exclude vascular puncture, 0.5 ml kg−1 of 0.25% bupivacaine solution was injected (Figure 1).

### 2.1.2. QL block

**Figure 2:**
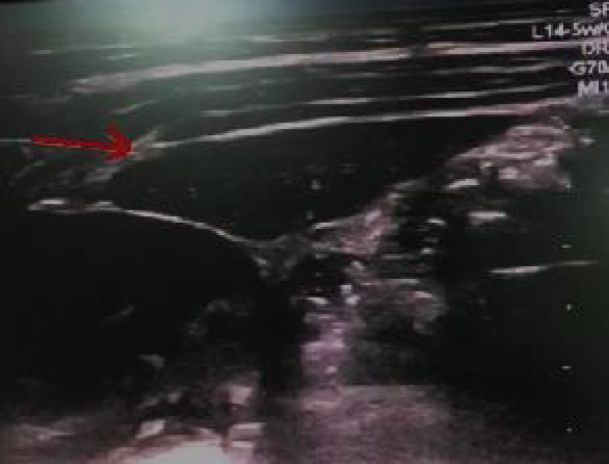
QL block.

After induction of anesthesia in the supine position, a 14-5 MHz linear ultrasonography probe was placed between the iliac crest and costal margin. The external oblique, internal oblique, and transversus abdominis muscles were identified, the probe was moved posteriorly and the QL muscle was visualized, and the midline of the thoracolumbar fascia was visible as a bright hyperechogenic line. The probe was attached to the area of the triangle of Petit until the QL was confirmed. A 22-gauge, 50-mm needle tip was placed at the anterolateral border of the QL following a negative aspiration of blood, then 0.5 ml kg−1 of 0.25% bupivacaine was injected between the QL muscle and the thoracolumbar fascia (lateral QL block approach) (Figure 2).

### 2.1.3. CEB

**Figure 3:**
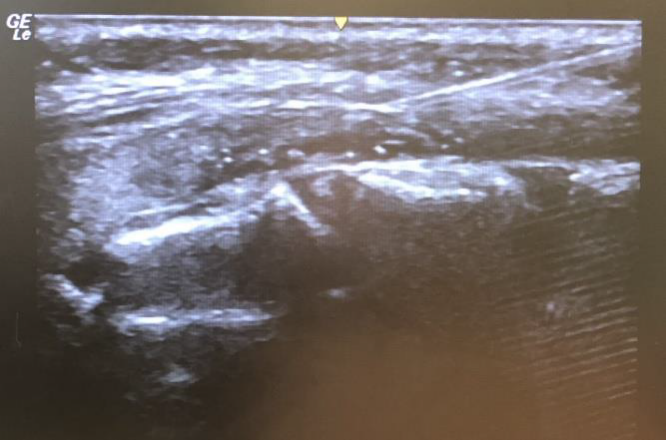


After induction of anesthesia, each patient was placed in the left lateral decubitus position. Under aseptic precautions, a 14-5 MHz linear ultrasound probe was placed on to the sacrococcygeal region. Dura mater, epidural space, conus medullaris, sacral cornua, and sacrococcygeal ligament were identified. Using the in-plane technique, a 25-gauge, 30-mm bevelled needle (B. Braun Melsungen, EpicanPaed caudal) was introduced to reach the sacral epidural space and 0.5 ml kg−1 of 0.25% bupivacaine solution was injected carefully after negative aspiration (Figure 3).

After administration of the nerve blocks, heart rate, blood pressure, SpO2, and BIS levels were monitored every 5 min until surgical incision, followed by monitoring at 10-min intervals until recovery from anesthesia. End-tidal sevoflurane concentration and additional opioid requirements were also recorded.

After the operations, duration of surgery was noted and the patients were transferred to the recovery room. Patients were observed for 30 min in the recovery room, and then sent to the ward. When the children were awake, Pediatric Objective Pain Scale (POAS), ventilatory frequency, arterial pressure, and heart rate were documented by a blinded investigator.

Pain assessments were made by using POAS at 0.5, 1, 2, 4, 8, 12, and 24 h after recovery from anesthesia (Table 1). If the POAS was greater than 5 in the recovery room and the surgical ward, IV paracetamol 10 mg kg−1 was administered. The same dose was repeated if needed after 30 min. Nursing staff that were to administer rescue analgesia on the surgical ward were blind to the group allocation of patients. Parents were informed about pain assessment and were instructed to give 10 mg kg−1 of ibuprofen syrup to their children if they experienced pain at home.

**Table 1:** Pediatric Objective Pain Scale (POAS).

Criteria		Points
Blood pressure	+10% of preoperative10%–20% of preoperative20%–30% of preoperative	012
Crying	Not cryingCrying but responding to tender loving careCrying but does not respond to tender loving care	012
Moving	NoneRestlessThrashing	012
Agitation	Patient asleep or calmMildHysterical	012
Verbal evaluation	Patient asleep or states no painMild pain (cannot localize)Moderate pain (can localize verbally or by pointing)	012

Further records compiled 24 h after the operation included time of first analgesic requirement; total analgesics consumption; length of hospital stay; adverse effects including nausea, vomiting, hypotension, motor weakness, and urinary retention; and satisfaction levels of the patients’ parents and of the surgeons.

### 2.2. Statistical analysis

Statistical analyses were performed by using IBM SPSS Statistics 22 (IBM SPSS, Turkey). Conformance of the variables with normal distribution was measured by using the Shapiro–Wilks test. In addition to descriptive statistics (average, SD, etc.), normally distributed variables were compared using a one-way ANOVA analysis of variance test. In the comparison of quantitative findings among groups, Tukey’s Honest Significant Difference test was used to determine the group causing the difference. Intergroup variables without normal distribution were compared using the Kruskal–Wallis test, and the group causing the difference was identified via the Mann–Whitney U test. The comparison of the normally distributed quantitative data within the groups was performed using the Paired-Sample t-test, and intragroup variables without normal distribution were compared using the Wilcoxon Signed Rank test. Qualitative data were tested using the chi-square and Fisher–Freeman–Halton test. P < 0.05 was considered statistically significant.

Sample size was calculated based on data from the study by Sahin et al. [12]. Twenty-five subjects were required to detect differences in total rescue analgesia requirement doses; that is 0.373 (140), power: 0.80 and 0.05. Due to the possibility of some subjects being excluded, the study was planned to include 35 patients in each group.

## 3. Results

Six patients from the TAP block group and 5 patients from the CEB group were excluded as their parents requested circumcision for their children. The study included a total of 94 patients. The groups were comparable based on age, sex, weight, ASA scores, and operation type (Table 2).

**Table 2:** Comparison of demographic and clinical data between the groups.

	Group			P
	TAP (n: 29)	QLB (n: 35)	Caudal (n: 30)	
	Mean ± SD	Mean ± SD	Mean ± SD	
Age (years)	4.16 ± 2.55	3.89 ± 3.26	2.99 ± 2.66	10.234
Weight (kg)	17.93 ± 10.93	16.74 ± 8.87	13.91 ± 7.5	10.226
Sex (M/F), n (%)				
F	10 (34.5%)	7 (20%)	3 (10%)	20.070
M	19 (65.5%)	28 (80%)	27 (90%)	
ASAn (%)				
1	23 (85.2%)	34 (97.1%)	25 (83.3%)	20.150
2	4 (14.8%)	1 (2.9%)	5 (16.7%)	
Operation type, n (%)				
Hydrocelectomy	1 (3.4%)	7 (20%)	2 (6.7%)	20.081
Inguinal hernia	21 (72.4%)	22 (62.9%)	15 (50%)	
Orchiopexy	6 (20.7%)	6 (17.1%)	12 (40%)	
Orchiopexy+hydrocelectomy	0 (0%)	0 (0%)	1 (3.3%)	
Orchiopexy+İng. hernia	1 (3.4%)	0 (0%)	0 (0%)	

1One-way ANOVA Test 2Chi-square test *P < 0.05Values are mean ± SD: standard deviation or n (%)

### 3.1. Intraoperative period

The time required to perform CEB was longer than that required for TAP or QL block (P < 0.05). Duration of anesthesia did not prolong duration of surgery (Table 3). There were no statistically significant differences among the groups with regard to heart rate, blood pressure, SpO2, and BIS levels during the intraoperative periods (P > 0.05). Fentanyl requirements and end-tidal sevoflurane concentration did not differ among the groups during intraoperative periods (P > 0.05).

**Table 3:** Comparison of perioperative period parameters between the groups.

	Group			P-value	TAP (n:29)	QLB (n:35)	Caudal (n:30)	Mean ± SD	Mean ± SD	Mean ± SD
	Time to first request analgesic (h)	1.54 ± 0.63 (1.8)	2.17 ± 1.94 (1.5)		5.08 ± 5.71 (3.3)	10.486
	Rescue analgesia requirement dosage (mg)	350 ± 173.21 (300)	270.83 ± 146.98 (225)		158.33 ± 49.16 (150)	10.046*
Rescue analgesia requirement rate n (%)				
Yes	4 (13.8%)	6 (17.1%)	6 (20%)	20.818
No	25 (86.2%)	29 (82.9%)	24 (80%)	
Discharge time	7.93 ± 4.08 (6)	6.4 ± 3.16 (6)	8.93 ± 5.57 (7)	10.006*
Complication n (%)				
Yes	5 (17.2%)	1 (2.9%)	3 (10%)	30.125
No	24 (82.8%)	34 (97.1%)	27 (90%)	

1The Kruskal–Wallis test 2The Fisher–Freeman–Halton test *P < 0.05*P < 0.05 is statistical significance, Values are mean ± SD: standard deviation or n (%)

### 3.2. Postoperative period

 The need for additional analgesia was significantly higher in the TAP block group than in the other groups (P < 0.05). Additional mean paracetamol requirements were 300 mg, 225 mg, and 150 mg in the TAP block, QL block, and CEB groups, respectively (P < 0.05).

**Figure 4:**
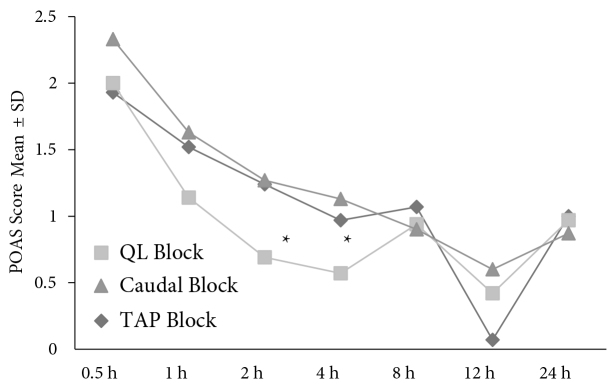


The POAS scores from the second and fourth hour in the QL block group were significantly lower than those in the other groups (P < 0.05) (Figure 4). 

No postoperative difference in vital signs was observed in the groups. There was no statistically difference in initial analgesic requirement, total analgesics consumption, and adverse effects among the groups (P > 0.05) (Table 4). Two postoperative patients in the caudal block group were unable to stand during the first 2.5 h (2.57 ± 0.99)**. **Postoperative urinary retention was also noted in 3 patients in the caudal block group. There was a statistically significant difference in length of hospital stay in the caudal block group compared to the QL block group (P < 0.05). There was no statistically significant difference among the groups in terms of parent and surgeon satisfaction levels (P > 0.05).

**Table 4:** Comparison of postoperative period parameters between the groups.

	Group			P-value	TAP (n:29)	QLB (n:35)	Caudal (n:30)	Mean ± SD	Mean ± SD	Mean ± SD
	Time to first request analgesic (h)	1.54 ± 0.63 (1.8)	2.17 ± 1.94 (1.5)		5.08 ± 5.71 (3.3)	10.486
	Rescue analgesia requirement dosage (mg)	350 ± 173.21 (300)	270.83 ± 146.98 (225)		158.33 ± 49.16 (150)	10.046*
Rescue analgesia requirement rate n (%)				
Yes	4 (13.8%)	6 (17.1%)	6 (20%)	20.818
No	25 (86.2%)	29 (82.9%)	24 (80%)	
Discharge time	7.93 ± 4.08 (6)	6.4 ± 3.16 (6)	8.93 ± 5.57 (7)	10.006*
Complication n (%)				
Yes	5 (17.2%)	1 (2.9%)	3 (10%)	30.125
No	24 (82.8%)	34 (97.1%)	27 (90%)	

1The Kruskal–Wallis test 2The Fisher–Freeman–Halton test *P < 0.05*P < 0.05 is statistical significance, Values are mean ± SD: standard deviation or n (%)

## 4. Discussion

We concluded that, while the amount of opioid consumed during intraoperative periods of perineural and caudal epidural (TAP and QLB) blocks is similar, in the postoperative period QLB is more beneficial to the recovery profile of pediatric patients.

The result of the present study confirm that ultrasonography has become an additional routine guide, especially in perineural blocks, and that minimally invasive analgesic methods have begun to replace central blocks in the perioperative periods [13].

Caudal block is the preferred regional anesthesia technique in pediatric patients [14]. Although the caudal block provides perfect analgesia, there is a risk of neurological complications. For this reason, practitioners are exploring other analgesic methods [15]. In our study, these results were supported by the initial dose of analgesic being administered at the fifth hour following surgery in the caudal block group and a score of less than 3 on the Pediatric Objective Pain Scale during 24 h postoperative period. However, 2 patients in the caudal block group suffered an average of 2.5 h of motor weakness and 3 events of urinary retention, which prolonged the discharge time. We think that this result does not change parent and surgeon satisfaction levels because of the small sample size. The caudal blocks were also associated with longer blockade and anesthesia periods than the other 2 groups. TAP and QL block were performed in the supine position, while caudal block was applied in lateral decubitus position. Image adjustment with USG and use of a different needle from other applications may also cause the caudal block to take longer.

Studies indicate that the analgesic quality of the TAP block renders it a viable alternative to the central nerve block [16]. In a recently published study, the TAP block and caudal block were compared in children undergoing lower abdominal surgery. It showed that firstly, the median duration of postoperative analgesia was significantly greater (3.5 h vs 6 h) in children who received CEB; secondly, there was no difference in the rescue analgesia requirements between the groups; thirdly, children who received CEB experienced greater incidence of pain in the 6 to 24 h postoperative interval; and finally, the number of children requiring rescue analgesia in the first 24 h postoperatively was significantly lower in the TAP group [16]. In another study, Sahin et al. compared the efficacies of the caudal block, ultrasound-guided TAP block, and ilioinguinal/iliohypogastric (II/IH) blocks for postoperative analgesia in children undergoing lower abdominal surgery. Caudal and TAP blocks were found to provide comparable postoperative analgesia, total analgesic consumption, and time to first analgesic requirement [12]. In our study, we found that TAP block was not as effective as caudal block or QL block. In 4 patients from the TAP group, effective analgesia was achieved only after additional paracetamol. The same patients were given oral ibuprofen at home. In a cadaver study by Elsharkawy et al., it is stated that the mechanism of analgesia may not solely involve blockade of distal sensory efferents, but may be due to a more proximal effect, perhaps at the level of the paravertebral space [17]. Therefore, it remains unclear whether TAP techniques result in LA spread into the PVS.

QL block is a new abdominal truncal block used for somatic analgesia of both the upper and lower abdomen [14]. QL block has an excellent analgesic effect on pain reduction, with patients reporting a reduction of 1–2/10 on the pain scale, usually lasting more than 24 h. Aksu et al. initiated ultrasound-guided QL block to provide postoperative analgesia for ambulatory surgeries in pediatric anesthesia practice. They presented results from their first 10 patients. The patients were observed as relaxed and calm in the postoperative care unit. None of the patients required additional analgesics. Patients were discharged from hospital at postoperative fourth and fifth hour. They admit that controlled studies involving a sufficient number of patients are required in order to detect the distribution of the local anesthetics and the field of coverage [18].

In a recent study, Oksuz et al. compared the efficacy of the TAP and QL block for postoperative analgesia in lower abdominal surgeries. It was reported that the number of patients who required analgesia in the first 24 h following surgery was significantly lower in the QL block group than in the TAP block group. In the QL block group, the postoperative 30 min and 1, 2, 4, 6, 12, and 24 h FLACC (Face, Legs, Activity, Cry, Consolability) scores were lower compared to those of the TAP block group. The first analgesic request time was 15 h in the QL block group and 10 h in the TAP group [19]. In our study, it was observed that QL block provided lower postoperative pain scores and shorter periods of hospital stay. However, the first analgesic request time and number of patients who required analgesia in the first 24 h postoperatively was not significantly different between the groups. The true mechanism of analgesia provided by QL block has not yet been fully clarified. It is believed that the local anesthetics spread in a segmental longitudinal pattern, and the endothoracic fascia into the paravertebral space. Therefore, the assumption is that visceral analgesia results from the spread of anesthetics to the celiac ganglion or sympathetic trunk via splanchnic nerves, as is the case with the paravertebral block [20].

In a small number of pediatric studies with QL block and TAP block, duration of postoperative analgesia, the rescue analgesia requirements, and the number of patients requiring analgesia during the first 24 h were reported differently [12,16,18,19]. These results are each different from each other and can only be explained by only the distal sensory efferents distribution or by the paravertebral space distributions of the local anesthetics.

In conclusion, in our study of pediatric patients who underwent lower abdominal surgery, we have found that TAP block caused higher additional analgesic consumption, caudal block led to prolonged hospital stays, and QL block provided lower postoperative pain scores. We suggest that ultrasound-guided QL block could be considered as an option for perioperative analgesia methods in pediatric patients undergoing lower abdominal surgery if the expertise and equipment are available.
